# Publisher Correction: Club-like receptors respond to light touch but not to whisking

**DOI:** 10.1038/s41467-026-69653-0

**Published:** 2026-02-17

**Authors:** Taiga Muramoto, Takahiro Furuta, Taro Koike, Knarik Bagdasarian, Sotatsu Tonomura, Aya Takenaka, Yosky Kataoka, Mitsuyo Maeda, Asami Eguchi, Masaaki Kitada, Kenzo Kumamoto, Ehud Ahissar, Satomi Ebara

**Affiliations:** 1 Department of Anatomy, Meiji University of Integrative Medicine, Nantan, Kyoto Japan; 2 Department of Systematic Anatomy and Neurobiology, Graduate School of Dentistry, The University of Osaka, Suita, Osaka Japan; 3 Department of Anatomy, Kansai Medical University, Hirakata, Osaka Japan; 4 Department of Brain Sciences, Weizmann Institute of Science, Rehovot, Israel; 5 Department of Anatomy, Kawasaki Medical School, Kurashiki, Okayama Japan; 6 Graduate school of Science, Technology and Innovation, Kobe University, Kobe, Hyogo Japan; 7 Laboratory for Chemical Biology, RIKEN Center for Biosystems Dynamics Research (BDR), Kobe, Hyogo Japan; 8 Japan Electron Optics Laboratory (JEOL) Ltd., Akishima, Tokyo Japan

**Keywords:** Whisker system, Touch receptors

Correction to: *Nature Communications* 10.1038/s41467-025-67514-w, published online 24 December 2025

In Fig. 1a of this article the tip of the glass pipette was incorrectly depicted as elongated and sharply pointed, penetrating the trigeminal tract; the original and corrected figure are shown below.

Incorrect Fig. 1
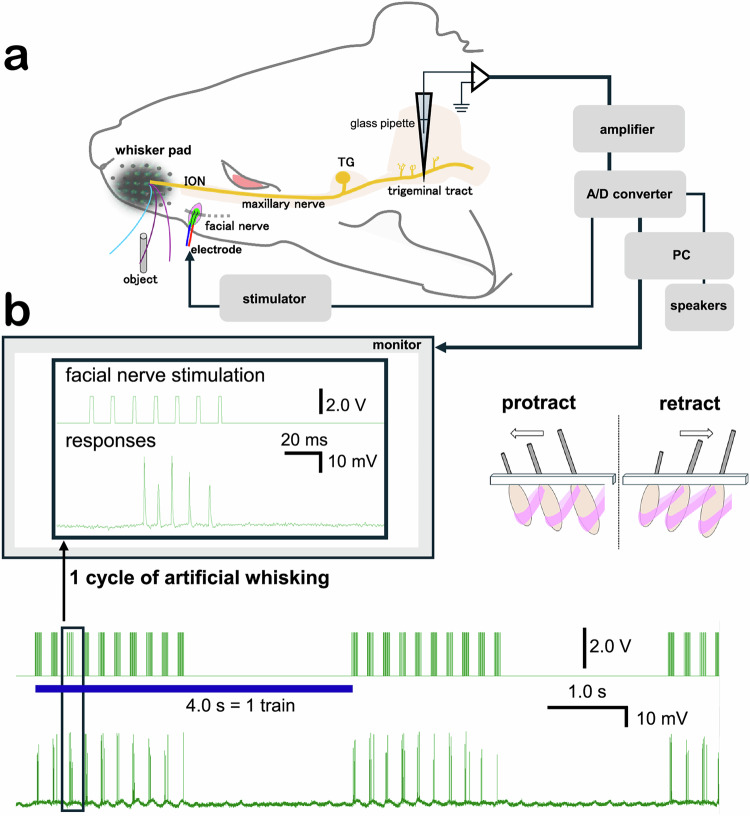


Correct Fig. 1
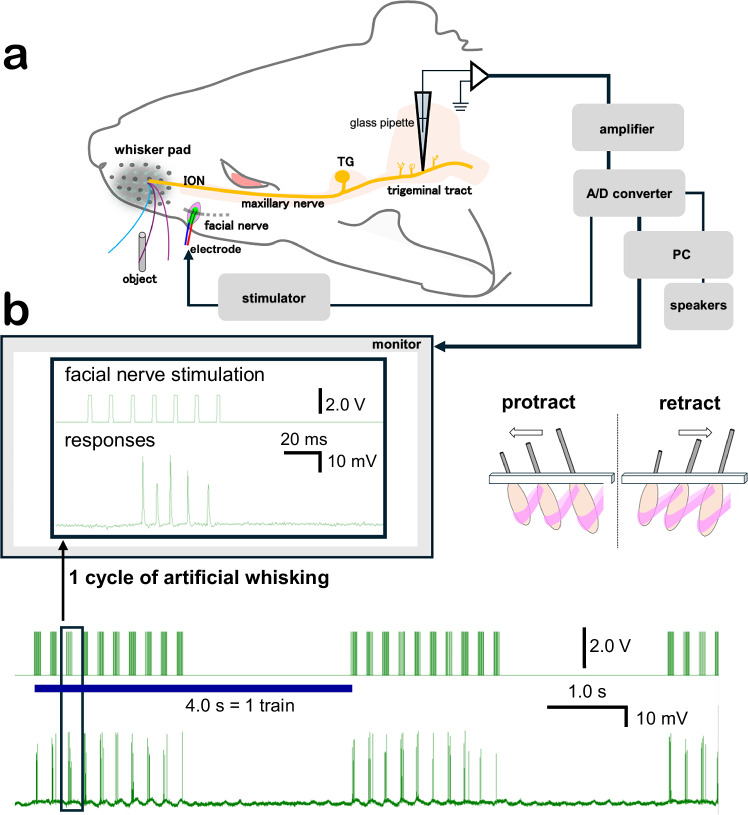


The original article has been updated.

