# Supplementary material for: Club-like receptors respond to light touch but not to whisking
Source: Nat Commun. 2025 Dec 24;16:11343. doi: 10.1038/s41467-025-67514-w (PMC12738765; doi:10.1038/s41467-025-67514-w)
Supplement: Supplementary file 1 — Supplementary Information [file 41467_2025_67514_MOESM1_ESM.pdf]

TITLE

Club-like Receptors Respond to Light Touch but not to Whisking

SUPPLEMENTARY INFORMATION

Supplementary Figure 1.

Supplementary Figure. 2.

Supplementary Methods.

Supplementary Fig. 1a.

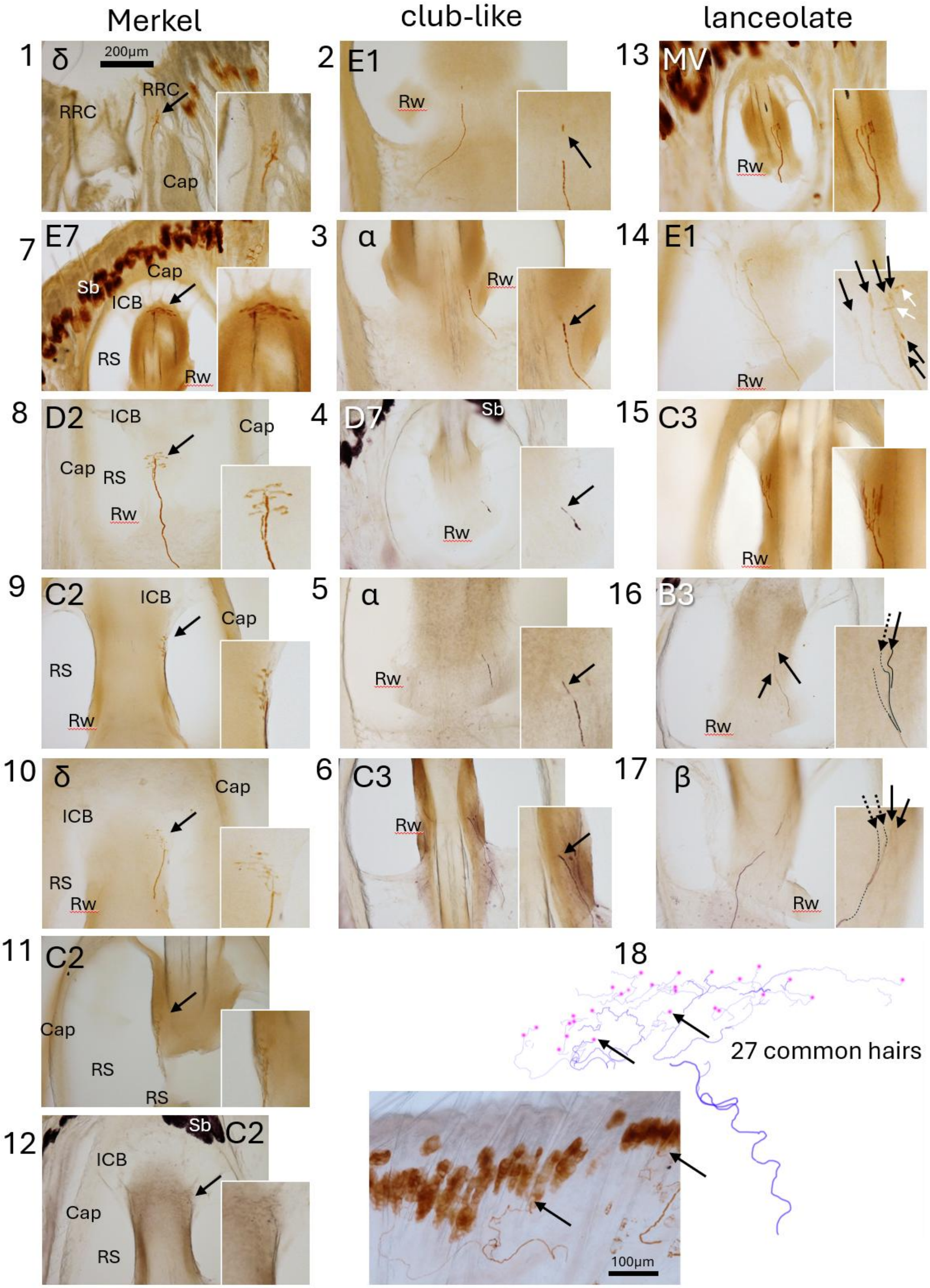

Supplementary Fig. 1b.

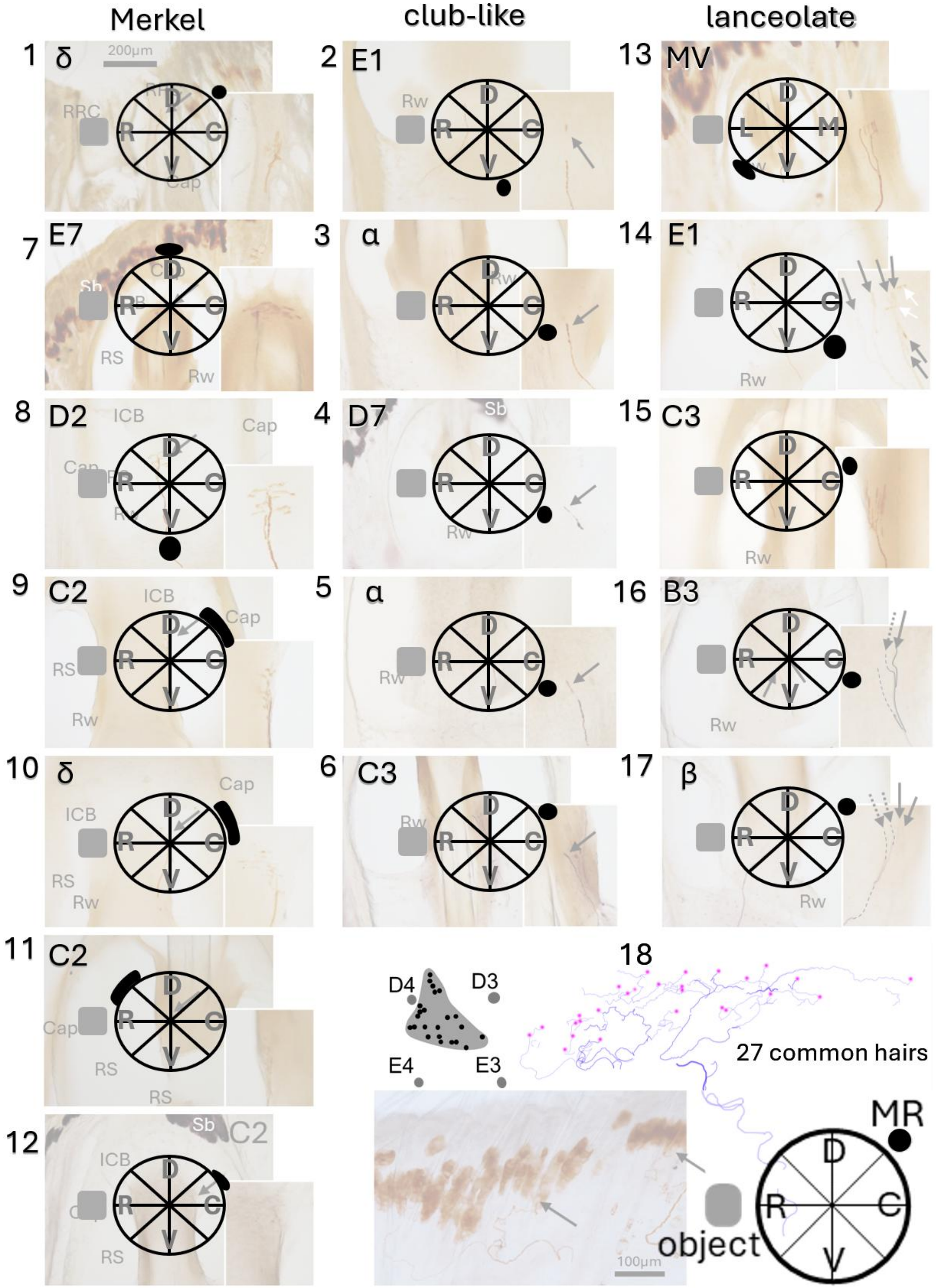

Supplementary Figure 1. Labeled mechanoreceptors.

1a: All 18 neurons identified mechanoreceptor types by morphology of labeled endings on the responded vibrissal follicles. Only No.18 was on 27 common hairs around 2 whiskers. Next to each figure is a 4x magnified photograph of the traced receptor (arrows). Dashed arrows indicate a part of mechanoreceptors that were on the next serial sections. White arrows were deposits. Cap: capsule, Sb: sebaceous gland.

1b: Azimuthal position of the receptors. D, dorsal; R, rostral; V, ventral; C, caudal; L, lateral; M, medial. Black dot or elongated shape indicate the location of the endings. Gray square indicates object azimuth.

Supplementary Figure 2.

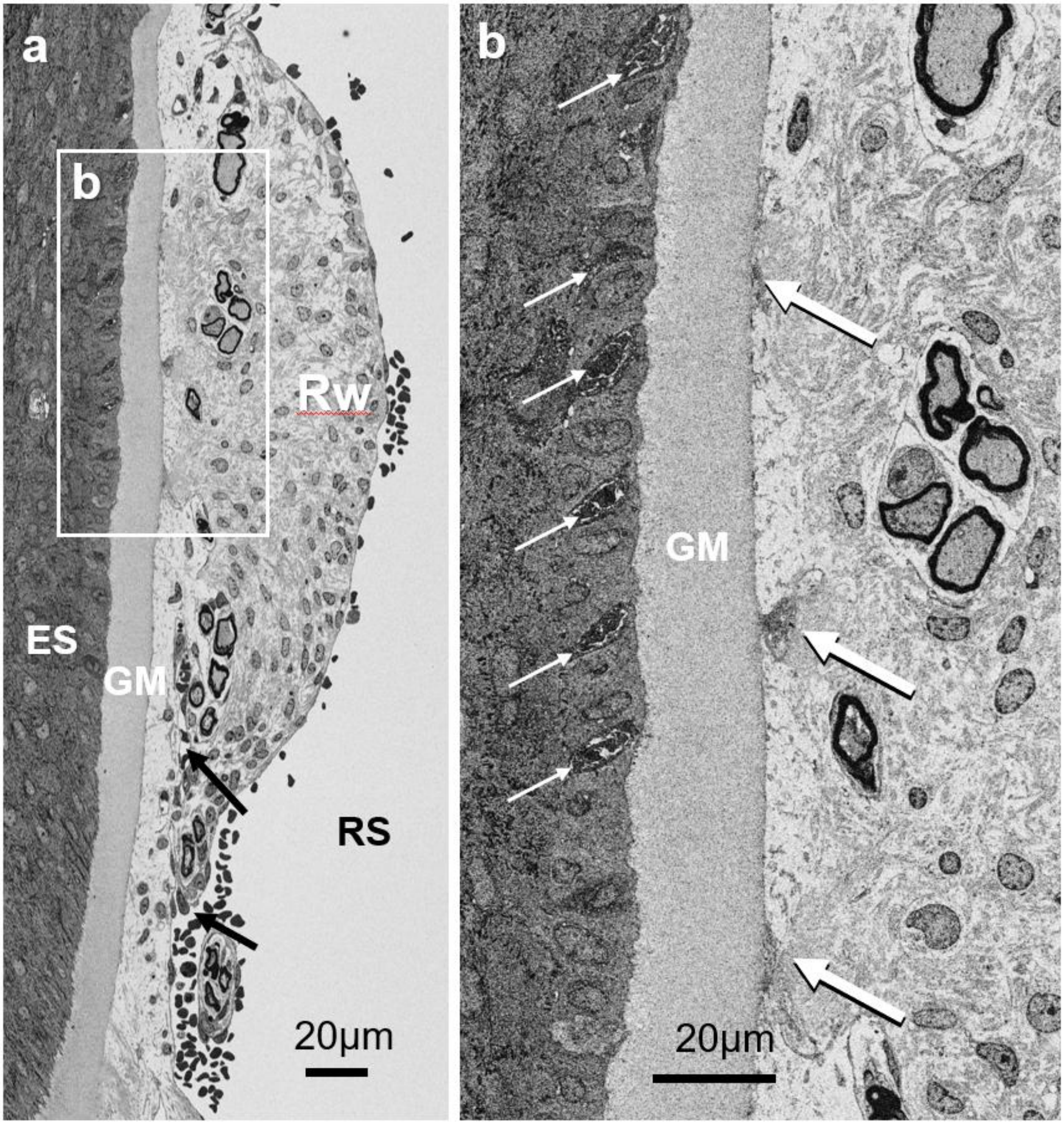

Supplementary Figure. 2. Ultrastructure of the cat Rw. a: The Rw of the cat is flat and not very thick, there is no curve in the ES, and the continuation of the RS is not visible from below (arrows). The GM is thick and flat, and the Rw shows no thick collagen bundles. **b:** Enlarged picture shows three club-like endings (large white arrows) attached to the thick GM. The endings are enclosed fine collagen fibers. Remarkably, Merkel terminal disks (small white arrows) on Merkel cells also were observed at the level of the Rw.

Supplementary Methods.

Cat samples obtained in previous studies (Ebara et al., 2002, 2003) were reprocessed and analyzed. These cats (1-3 years old, both sexes, mongrel, house-bred) were properly cared for, maintained, and used in accordance with the regulations of the Meiji University of Oriental Medicine Animal Care and Use Committee at the time. Vibrissal follicles were dissected after perfusion with fixative under deep anesthesia and embedded in Epon blocks using the same procedure as described in this main text. In this study, three appropriate blocks containing vibrissal follicles were processed for array tomography and observed.
