# Supplementary material for: Club-like receptors respond to light touch but not to whisking
Source: Nat Commun. 2025 Dec 24;16:11343. doi: 10.1038/s41467-025-67514-w (PMC12738765; doi:10.1038/s41467-025-67514-w)
Supplement: Supplementary file 2 — Description of Additional Supplementary Files [file 41467_2025_67514_MOESM2_ESM.pdf]

Description of Additional Supplementary Files

File name: Supplementary Movie 1
Description: Reconstruction of adjacent two club-like endings and surrounding tissues.

File name: Supplementary Movie 2
Description: Paper model illustrating the whisking-response-damping hypothesis. This video shows a simple thick-paper-based mechanical model demonstrating how vibration is differentially transmitted depending on structural support. Two elevated platforms receive floor vibrations, but only one supports an inertial mass. The green plastic cap on the damped platform moves less, indicating effective vibration damping. This setup is used to illustrate our hypothesis: the inertial mass represents the ringwulst (black circle with red bar), the yellow triangle corresponds to club-like mechanoreceptor endings, the sky-blue surface represents the glassy membrane, and the dark-blue base symbolizes the follicle's capsule. Movement of the capsule—analogous to whisker follicle motion during active whisking—is transmitted via a green dashed strip, modeling connective tissue. The reduced movement of the green plastic cap exemplifies how the ringwulst might dampen whisking-related vibrations, allowing club-like endings to selectively respond to touch.
